# Book Reviews

**Published:** 1894-01

**Authors:** 


### Book Reviews.

Minor Surgery and Bandaging. By Henry R. Wharton, M. D., Demonstrator of Surgery in the University of Pennsylvania. In one 12mo volume of 529 pages, with 416 engravings, many being photographic. Cloth, $3.00. Philadelphia: Lea Brothers & Co. 1893.

It is but little more than two years ago that we published a review notice of Wharton's first edition. At that time, we remarked that the book was one of the very best treatises on minor surgery that had been published, that it ought to be adopted as a text-book on the subjects of which it treats, and that it contained more practical surgery within its limits and boundaries than any book of its kind we had ever seen. What was true of the first edition may be, with propriety, repeated and accentuated in regard to this second and revised edition. Its illustrations are to be specially commended, particularly those that relate to bandaging, most of which have been taken from photographs of applied bandages in the several localities of the body. The author has thoroughly revised that portion of the work relating to the aseptic and antiseptic methods of wound treatment, than which there is no more important subject in the whole domain of surgery. Much new matter has been added, which brings it abreast of the very latest knowledge on the subjects of which it treats. It is printed and bound in exceptionally handsome style, and will easily find its way into the libraries of physicians and to the tables of students.

A Manual of Medical Treatment, or Clinical Therapeutics. By I. Burney Yeo, M. D., F. R. C. P., Professor of Therapeutics in Kings' College, London. In two 12mo volumes, containing 1275 pages, with illustrations. Complete work, cloth, $5.50. Philadelphia: Lea Brothers & Co. 1893.

This work is unusual, in that it is devoted exclusively to the treatment of disease,—symptomatology, pathology, diagnosis, and other questions being considered only when necessary to touch upon them with reference to elucidating therapeutic management. Yeo is especially strong with reference to the treatment of cardiac affections, and he accentuates cardiac neuroses in a clear and interesting manner. At the end of each subject are placed formulæ, additional to those in the text, that may prove useful in the treatment of the disease under consideration. The work is especially well indexed, and the size of the volumes makes them convenient for reference. It is impossible, without making such a work too voluminous, to cover the entire field of medicine in every detail, but Yeo has succeeded in making a treatise that will easily fall into the hands of progressive physicians as a manual of treatment.

---

DUANE'S STUDENTS' DICTIONARY OF MEDICINE. The Students' Dictionary of Medicine and the Allied Sciences. Comprising the pronunciation, derivation and full explanation of medical terms, together with much collateral descriptive matter, numerous tables, etc. By ALEXANDER DUANE, M. D., Assistant Surgeon to the New York Ophthalmic and Aural Institute; Reviser of Medical Terms for Webster's International Dictionary. In one square octavo volume of 658 pages. Cloth, $4.25; half leather, $4.50; full sheep, $5.00. Philadelphia: Lea Brothers & Co. 1893.

Good medical dictionaries are becoming plentiful, but not so with reference to those that are especially adapted to the use of students. The latter must be equipped with a dictionary that contains information concerning every word that he will be likely to meet with in his daily studies, and, at the same time, be sufficiently compact as to be easily handled. Such dictionaries, of course, must have in view practical utility rather than complete philological accuracy or historical tradition. Duane claims to have constructed his work on this plan, and we are inclined to the opinion that he has succeeded most satisfactorily. The work consists, as he states in his introduction, of a series of major titles or primes, each beginning a separate paragraph and printed in heavy type. The arrangement of the main headings is strictly alphabetical, while the system of spelling adopted is intended to indicate the best usage, regardless of analogy. His use of the hyphen is to be commended. He omits it in compound expressions, except when employed to separate two vowels which might otherwise be regarded as forming a diphthong, and also when used to connect two words of coördinate importance. We differ from the author in his spelling in some instances, because we think that every effort should be made by constructors of dictionaries to reduce the spelling of medical terms to the utmost simplicity consistent with an adherence to roots, prefixes, and affixes. In pronunciation we have much praise to bestow upon this author, for he is more accurate in this line than most of his contemporaries. We regard it essential to teach students a uniform, correct, and finished pronunciation, for it is fast becoming one of the ways in which medical scholarship is to be recognized. An accurate pronunciation and use of good English is everywhere considered a requisite in good society. So, too, will, sooner or later, good medical pronunciation be considered a requisite for membership in polite medical circles. We presume that this book will find its way into the libraries of good medical scholars without much urging.

---

Weekly Abstract of Sanitary Reports. Issued by the Supervising Surgeon-General, M. H. S., under the National Quarantine Act of April 29, 1878. Volume VII., Nos. 1 to 53. Washington: Government Printing Office. 1893.

This is a bound volume of the weekly reports issued by the Supervising Surgeon-General of the Marine Hospital Service. Each weekly report contains a summary of weekly inspections of immigrants at various points; reports of States and yearly and monthly reports of cities; a mortality table of the cities of the United States; table of temperature and rainfall, and, in addition, a summary of foreign reports received through the Department of State and other channels, and a mortality report of foreign cities. It is a most valuable volume for reference, and every sanitarian, health officer, and scientist should obtain the book.

---

Anatomy, Descriptive and Surgical. By Henry Gray, F. R. S., Lecturer on Anatomy at St. George's Hospital. London. New American from the thirteenth enlarged and improved English edition. Edited by T. Pickering Pick, F. R. C. S., Examiner in Anatomy, Royal College of Surgeons, of England. In one imperial octavo volume of 1100 pages, with 635 large engravings. Price, with illustrations in colors, cloth, $7.00; leather, $8.00. Price, with illustrations in black, cloth, $6.00; leather, $7.00. Philadelphia: Lea Brothers & Co. 1893.

This is an old friend in a new dress. Every student of medicine since 1858 has been familar with Gray's Anatomy. The author originally constructed his work with reference to its importance to the surgeon, and so introduced under each subdivision observations on practical points of surgery with reference to the part under examination. The editor has followed, in this edition, the original lines marked out by Gray, thus keeping prominent the fact that the work is intended for students of surgery rather than for the scientific anatomist. The whole work has undergone careful revision, so as to bring it abreast of modern teachings in anatomy. We always had a kindly regard for the illustrations in Gray, where each organ, tissue, artery, and nerve bear their respective names, and in this edition color has been worked to advantage in bringing out the relationship of vessel and nerve. Of late years, many works on anatomy have been introduced to the profession, but, as a reference book for the practical everyday physician, and as a text-book for the student, we think it will be difficult to supplant Gray.

---

A Handbook of Ophthalmic Science and Practice. By Henry E. Juler, F. R. C. S., Ophthalmic Surgeon to St. Mary's Hospital; Surgeon to the Royal Westminster Ophthalmic Hospital; Consulting Ophthalmic Surgeon to the London Lack Hospitals. With illustrations. Second edition. Philadelphia: Lea Brothers & Co. 1893.

The second edition of this handbook is a decided improvement upon the first edition. Numerous additions have been made, excellent illustrations serve to make plain the subject matter, and the work generally has been greatly improved and brought down to date. Modern accepted methods of treating the various diseases of the eye are described. The chapter on the refraction of the eye is excellent, and contains a very clear explanation of the principles involved in retinoscopy. The author has not neglected to give some space to the consideration of muscular insufficiencies, although the subject is merely introduced, and no discussion of it is attempted. This is to be regretted, inasmuch as this subject is one which is daily assuming more and more importance. On the whole, the book is a valuable contribution to ophthalmic literature, and will be referred to with confidence. S.

---

The Physicians' Visiting List (Lindsay & Blakiston) for 1894. Forty-third year of its publication. Sold by all booksellers and druggists. Philadelphia: P. Blakiston, Son & Co., 1012 Walnut street.

This is an old friend, and a most welcome one. It contains almost everything that can possibly be required by the busy physician in his pocket visiting list, among which may be mentioned a table of signs; the metric system; table for converting apothecaries'

weights and measures into grams ; a posological table ; a dose table ; a list of new remedies ; incompatibilities, poisons, and antidotes, disinfectants ; notes on the examination of urine ; information of Bright's diseases ; diagnosis and treatment of the simpler superficial diseases of the eye ; asphyxia and apnea ; comparison of thermometers, and a table for computing the period of utero gestation. Then follow the usual blank pages of the visiting list proper, arranged for twenty-five patients per week ; next, memoranda for the twelve months of the year; then, addresses of patients and others, afterward nurses' addresses ; then, blank pages for bills and accounts; obstetric engagements ; vaccination engagements ; record of obstetric cases ; records of deaths, and, finally, cash account.

It is bound in black morocco, and furnished with a Dixon pencil and eraser. We regard it as one of the most complete visiting lists in the market.

---

The Throat and Nose, and Their Diseases. By Lennox Browne, F. R. C. S. E., Senior Physician to the Central London Throat and Ear Hospital. Fourth and enlarged edition. In one imperial octavo volume of about 750 pages, with 120 illustrations in color, and 235 engravings on wood. Cloth, $6.50. Philadelphia: Lea Brothers & Co. 1893.

The author of this work is one of the masters of the laryngological art, and when he writes or speaks, it is because he has something of interest to bring to the attention of his professional colleagues. There has been two years' delay between the third and fourth editions of Browne's treatise, thus leaving it practically out of print for that length of time. The author takes advantage of this fact to plead that delays of this kind are not altogether without advantage to his readers. We are well aware that the science of laryngology is making such rapid strides that even in two years much of it has to be rewritten. Opinions that we then accepted as abounding in truth are now discarded altogether as valueless, or are sent forth in modified forms. The usual space in this work is devoted to the consideration of the anatomy and physiology of the throat and nose, after which the examination of the throat and larynx, with special reference to the use of the laryngoscope, is described. This is a most interesting chapter, as well as the next on the inspection of the mouth, fauces, and oro-pharynx, and chapter fourth on the laryngoscopic image. These, together with chapter five, on rhinoscopy and the rhinoscopic image, should be carefully studied by general practitioners of medicine with a view to gleaning intelligent information therefrom on the subject of diagnosis. The group of chapters beginning with six and ending with seventeen are devoted to a minute and detailed consideration of diseases of the throat, nose, mouth, pharynx, and larynx, and are, throughout, stamped with the author's personality. In support of his views he reports many clinical cases, that are printed in smaller type, but that forcibly illustrate the author's opinions on technical points. If there be those that differ from him on some of the propositions set forth in this book, they will yet find in it strong proof of the author's convictions on most points, and will find here a basis for an intelligent disagreement. Browne devotes a number of pages to the publication of formulæ that have proven valuable in nose and throat diseases, and finally are grouped, next before the index, fifteen colored plates contain ing 122 figures drawn by himself from Nature, and on stone.

---

A Manual of Diseases of the Ear. By George P. Field, M.R.C.S., Aural Surgeon and Lecturer on Aural Surgery, St. Mary's Hospital Medical School, London. In one octavo volume of 391 pages, with seventy-three engravings and twenty-one colored plates. Cloth, $3.75. Philadelphia: Lea Brothers & Co. 1893.

The popularity of this work is amply testified to in the fact that a fourth edition is now sought for by those who are interested in aural medicine. While this book is one of great value to the specialist, from which he may glean many profitable hints and established facts of value, it also has claims upon the observation of that portion of the profession who may not be strictly classed as specialists. The general practitioner must know something of the commoner ailments to which the ear is liable, particularly that he may make intelligent diagnosis. In reading Fields' chapters on the anatomy of the ear and the physiology of hearing, the general practitioner will refresh himself upon many forgotten points, and will even find new ones or those that he had heretofore overlooked. The text is especially clear and concise, and the illustrations accentuated beyond misunderstanding. So too with the chapter on examination of the patient, which, though brief, is one of the clearest and best we ever read on this subject. We shall not attempt an analysis of all the diseases of the ear that are here treated of or considered, but shall content ourselves with the general statement that it is one of the best of the smaller treatises and is a safe guide on the subject of which it discourses.

SURGERY. By BERN B. GALLAUDET, M. D., Demonstrator of Anatomy and Clinical Lecturer on Surgery, College of Physicians and Surgeons, New York ; Visiting Surgeon, Bellevue Hospital, New York; and Charles N. Dixon-Jones, M. D., Assistant Surgeon, Out-patient Department, Presbyterian Hospital, New York. Being the final volume of the Students' Quiz Series, edited by Bern B. Gallaudet, M. D. Duodecimo, 291 pages, 149 illustrations. Cloth, $1.75. Philadelphia : Lea Brothers & Co. 1893.

This volume completes the group of books known as the Students' Quiz Series, making altogether thirteen volumes. They are of uniform size, and are most handsomely printed and bound. This last volume, on Surgery, is more than a compend or question book, for it explains many points which are difficult of elucidation and still more difficult to understand. The author, who has had considerable experience in teaching surgery to students and graduates of medicine, has made an especial effort to bring out the difficult points of inflammation, septic infection, together with tubercular disease of bones, tumors, and cysts, brain and abdominal surgery. It is a little volume well worth its price.

---

THE ERA KEY TO THE UNITED STATES PHARMACOPEIA. A Complete List of the Drugs and Preparations of the United States Pharmacopeia, revision of 1890–93, giving official titles, common names and synonyms of the drugs, chemicals and preparations in the Pharmacopeia, with doses in apothecaries' weight and measures, with equivalents in metric terms. Compiled for the *Pharmaceutical Era*. Detroit, Mich. : D. O. Haynes & Co. 1893.

The publishers state that the object of this Key is to assist physicians and pharmacists to familiarize themselves with the contents of the new United States Pharmacopeia, also to further the introduction and employment of official drugs and preparations. We presume, from examining the little book, that it will be a useful one, and fill the purpose for which it is intended. It can be obtained of the publishers upon application, at the small price of twenty-five cents.

---

TRANSACTIONS OF THE FOURTEENTH ANNUAL MEETING OF THE AMERICAN LARYNGOLOGICAL ASSOCIATION, held in the city of Boston, June 20, 21, and 22, 1892. Octavo, pp. vi.—121. New York : D. Appleton & Co. 1893.

The annual volume of this Association is replete with excellent papers and good discussions. The present book is no exception to the rule. Dr. Beverly Robinson's paper reporting some cases of membranous sore throat elicited a spirited discussion. Two papers, one by Dr. W. H. Daly, and another by Dr. John O. Roe, relating to the technique of repairing broken noses, exhibits the marked improvements pertaining to that most unsightly deformity.

---

PHYSIOLOGY. By FREDERICK A. MANNING, M. D., Attending Surgeon, Manhattan Hospital, New York. Series edited by BERN B. GALLAUDET, M. D., Demonstrator of Anatomy, College of Physicians and Surgeons, New York. Visiting Surgeon, Bellevue Hospital, New York. Students' Quiz Series, No. 2. Pocket size, 12mo, 201 pages, sixty-nine illustrations, $1.00. Philadelphia: Lea Brothers & Co.

PRACTICE OF MEDICINE. By EDWIN T. DOUBLEDAY, M. D., Member of New York Pathological Society, and J. D. NAGEL, M. D., Member of New York County Medical Association. $1.00. Students' Quiz Series, No. 6. Philadelphia: Lea Brothers & Co.

These two books are a continuation of the group known as the Students' Quiz Series, and are uniform in style and price with the others that have been published. It seems to us that this group of books has been improperly named, for they contain something more than mere questions and answers. They go into considerable detail of explanation when it is essential to do so. In the present methods of teaching, some such system seems necessary, and this group, consisting of twelve or thirteen numbers, now published, admirably fills the place it is intended to occupy.

---

A TREATISE ON OPHTHALMOLOGY, for the General Practitioner. Second edition. Revised and enlarged, with 140 illustrations. By ADOLF ALT, M. D. St. Louis: J. H. Chambers & Co. 1893.

This work is not intended for the specialist, but for the general practitioner, to be a useful guide and help in caring for certain diseases of the eye. All the important affections of the eye are considered in a clear, concise manner, and numerous practical illustrations given. An important chapter for the general practitioner is that on the diagnostic value of eye diseases in intra-cranial affections. The work is a very acceptable addition to the literature of this class. S.

---

INTRODUCTION TO THE STUDY OF THE DISEASES OF THE SKIN. By P. H. PYE-SMITH, M.D., F. R. S., F. R. C. P., Physicion to the Department of Cutaneous Diseases in Guy's Hospital, London. In one handsome 12mo volume of 407 pages, with twenty-eight illustrations, eighteen of which are colored. Cloth, $2.00. Philadelphia: Lea Brothers & Co.

The chief aim of this treatise, as stated in the preface, is to bring the physician in closer contact with a more correct understanding of the local distribution of the diseases of the skin, by the introduction of wood-cuts, an improvement over the many other manuals which have recently appeared. In its preparation, the needs of the general practitioner have been kept primarily in view, the description being concise, yet containing a fund of useful knowledge not too theoretical or speculative. The simplest methods of treatment are indicated. The little volume is written in a style that will enable the student to understand well its various topics. The text is illustrated with twenty-six wood-cuts.

